# Impact of positive microscopic resection margins (R1) after gastrectomy in diffuse-type gastric cancer

**DOI:** 10.1007/s00432-023-04981-y

**Published:** 2023-06-21

**Authors:** Sérgio Gaspar-Figueiredo, Pierre Allemann, Alexander B. J. Borgstein, Gaëtan-Romain Joliat, Valentine Luzuy-Guarnero, Christophe Brunel, Christine Sempoux, Suzanne Sarah Gisbertz, Nicolas Demartines, Mark Ivo van Berge Henegouwen, Markus Schäfer, Styliani Mantziari

**Affiliations:** 1 Department of Visceral Surgery, Lausanne University Hospital CHUV, University of Lausanne, Rue du Bugnon 46, 1011 Lausanne, Switzerland; 2 Faculty of Biology and Medicine, University of Lausanne UNIL, 1011 Lausanne, Switzerland; 3 Department of Surgery, Amsterdam UMC Location University of Amsterdam, Amsterdam, The Netherlands; 4 Cancer Treatment and Quality of Life, Cancer Center Amsterdam, Amsterdam, the Netherlands; 5 Department of Pathology, Lausanne University Hospital and University of Lausanne, 1011 Lausanne, Switzerland

**Keywords:** Stomach neoplasms, Survival rate, Margins of excision, Recurrence, Drug therapy

## Abstract

**Introduction:**

Diffuse-type gastric cancer (DTGC) is associated with poor outcome. Surgical resection margin status (R) is an important prognostic factor, but its exact impact on DTGC patients remains unknown. The aim of this study was to assess the prognostic value of microscopically positive margins (R1) after gastrectomy on survival and tumour recurrence in DTGC patients.

**Methods:**

All consecutive DTGC patients from two tertiary centers who underwent curative oncologic gastrectomy from 2005 to 2018 were analyzed. The primary endpoint was overall survival (OS) for R0 versus R1 patients. Secondary endpoints included disease-free survival (DFS), recurrence patterns as well as the overall survival benefit of chemotherapy in this DTGC patient cohort.

**Results:**

Overall, 108 patients were analysed, 88 with R0 and 20 with R1 resection. Patients with negative lymph nodes and negative margins (pN0R0) had the best OS (median 102 months, 95% CI 1–207), whereas pN + R0 patients had better median OS than pN + R1 patients (36 months 95% CI 13–59, versus 7 months, 95% CI 1–13, *p* < 0.001). Similar findings were observed for DFS. Perioperative chemotherapy offered a median OS of 46 months (95% CI 24–68) versus 9 months (95% CI 1–25) after upfront surgery (*p* = 0.022). R1 patients presented more often early recurrence (< 12 postoperative months, 30% vs 8%, *p* = 0.002), however, no differences were observed in recurrence location.

**Conclusion:**

DTGC patients with microscopically positive margins (R1) presented poorer OS and DFS, and early tumour recurrence in the present series. R0 resection should be obtained whenever possible, even if other adverse biological features are present.

## Introduction

Gastric cancer newly affected over a million patients in 2018 and was responsible for at least 780,000 deaths, making it the third leading cause of cancer-related deaths worldwide (Bray et al. 2018). For practical reasons, the Lauren classification with its two types of gastric cancer is still often used. The diffuse-type gastric cancer (DTGC) is characterized by poorly cohesive cells, and the intestinal type typically shows gland-forming cells. During the past decades, the incidence of intestinal type has decreased, while a relative increase of DTGC was observed representing nowadays nearly 30% of all gastric cancer. Intestinal-type gastric cancer remains more frequent in older male patients, whereas DTGC is often seen in younger patients without clear sex predominance (Lauren 1965; Ikeda et al. 1995; Wu et al. 2009; Waldum and Fossmark 2018). Overall, DTGC patients have poorer prognosis than intestinal-type patients (Qiu et al. 2013; Petrelli et al. 2017).

Traditionally, 5-cm and 8-cm proximal safety margins are required for intestinal and diffuse-type to obtain R0 resection (Moehler et al. 2011; Japanese Gastric Cancer Association 2017; Mönig et al. 2020). In the case of DTGC, as submucosal invasion may extend particularly far from the macroscopic primary tumour site, intra-operative frozen-section analysis is recommended to increase R0 resection rates but without convincing results (Squires et al. 2014). Therefore, total gastrectomy has become the standard resection for almost all DTGC (Lee et al. 2014; Iii et al. 2015). The “5 cm and 8 cm” paradigm has recently been challenged, as the latest ESMO guidelines recommend 3 cm for intestinal type and 5 cm for diffuse type, respectively (Lordick et al. 2022). It has been suggested that the distance of the proximal resection margin was not a prognostic factor in itself, as long as microscopically negative margins (R0 resection) could be obtained (Ohe et al. 2014). Some authors even advocate that R1 resection may neither influence overall survival (OS) nor local recurrence (Ohe et al. 2014; Postlewait et al. 2015). This is especially true in advanced disease where the R status may be considered as a sign of aggressive tumour biology along with other adverse features, such as lymph node metastases, lympho-vascular and perineural invasion, and poor response to chemotherapy (Aurello et al. 2014; Lee et al. 2014; Ohe et al. 2014; Iii et al. 2015; Postlewait et al. 2015). A recent meta-analysis suggested that R1 margin patients should be reoperated only if lymph node yielded is poor (< 3 lymph nodes), whereas other studies do not support the need for revisional surgery to obtain complete surgical margins (Aurello et al. 2014; Postlewait et al. 2015).

Diffuse-type gastric cancer seems to have a poor response to systemic chemotherapy. Messager et al., in a series of 924 patients observed that perioperative chemotherapy (with traditional 5-FU-platin-based regimens) did not offer OS benefit or higher R0 resection rate in DTGC patients (Messager et al. 2011). Other studies suggested that once some histologic response is observed, DTGC patients also display some survival benefits (Heger et al. 2014; Pernot et al. 2015). The FLOT regimen was recently suggested offering better results in DTGC patients in terms of R0 resection and OS benefit (Chen et al. 2014; Hultman et al. 2014).

The aim of the present study was to assess the prognostic value of positive resection margins (R1) after oncologic gastrectomy, on survival and tumour recurrence in patients with diffuse-type gastric cancer.

## Methods

### Patients

All consecutive patients operated with curative intent for gastric cancer between January 2005 and December 2018 in the two participating tertiary centres (Lausanne University Hospital CHUV, Switzerland, and the Amsterdam UMC, location AMC, Amsterdam, Netherlands) were screened for possible inclusion. Only patients with DTGC [signet-ring cell carcinoma and other poorly cohesive carcinomas in WHO classification (Berlth et al. 2014)] were eligible for the present study. All included patients were > 18 years of age and provided written informed consent for research purposes. Patients with or without preoperative chemotherapy were eligible for inclusion. Patients with other histological types, macroscopically incomplete (R2) resection and distant metastases upon diagnosis were excluded, as well as patients with in-hospital 30-day mortality after the index operation.

The local ethics committee (CER-VD) approved this study [Protocol No 2018-00664], with an amendment to include data from the second university centre [2018-00664_am220301].

### Histopathological definitions

Patients with signet ring cell carcinoma and other poorly cohesive carcinomas were included as they correspond to the DTGC in the Lauren classification. Patients with mixed histological types were included if DTGC was the major, predominant component (> 75%) of the tumour. When no tumour was found in dissected lymph nodes in the pathological specimen, patients were considered (y-)pN0, whereas tumorous lymph node infiltration was considered as (y-)pN + . R1 status was defined as 0-mm distance between surgical resection margin and microscopic tumour limits, according to the American College of Pathologists’ definition ([Protocol for the Examination of Specimens From Patients With Carcinoma of the Stomach 4.2.1.0.pdf]. (2022)). Tumour stage was defined by the 8th TNM/UICC staging system (Liu et al. 2018). Peritoneal washing was not systematically performed in the two centres throughout the years, and if performed in its exact technique, the result assessment and therapeutic implications were not standardised. Thus, peritoneal results have not been included in baseline staging, to avoid confounding.

### Study endpoints

The primary endpoint was OS for R0 versus R1 patients. The lymph node status (pN0 vs pN +) was also taken into consideration to explore the specific impact of R1 resection in early versus locally advanced stages. Thus, four groups were created: pN0R0, pN + R0, pN0R1 and pN + R1. In addition, the impact of preoperative chemotherapy on OS was assessed. Secondary endpoints included disease-free survival (DFS), as well as recurrence patterns in terms of time-to-recurrence (early < 12 months versus late > 12 months), and location (local versus distant metastatic, including peritoneal carcinomatosis).

OS was calculated in months from the index surgery until the date of death or last follow-up. Long-term follow-up was last updated in January 2022 for all patients, through their electronic medical records, general practitioner, National civil registry or with direct contact with patients or families. If there was no event at the time of analysis, or if the patient was lost to follow-up, the case was censored. Recurrence was defined as the first recorded loco-regional (anastomotic, peritoneal, or regional lymph nodes) or distant (metastatic) tumoral relapse. DFS was estimated as the interval between index surgery and the first documented recurrence. Postoperative complications were recorded and classified according to the validated 5-scale Clavien system (Dindo et al. 2004).

### Statistical analysis

Discrete variables were compared with the chi-square or Fisher’s test as appropriate, whereas continuous variables were compared with the Mann–Whitney *U* test. Time-to-event outcomes (OS and DFS) were analysed with Kaplan–Meier method and compared with the log-rank test. A Cox proportional-hazards model was performed to assess variables independently predicting OS or DFS. Logistic regression analysis assessed predictors of loco-regional recurrence. Results are expressed as hazards ratio (HR) or odds ratio (OR) with 95% confidence interval (CI), and *p*-values < 0.05 were considered statistically significant. All statistical analyses were performed using SPSS Statistics software (version 25, IBM Corp, Armonk, NY, USA).

## Results

### Baseline and surgical characteristics

**Fig. 1:**
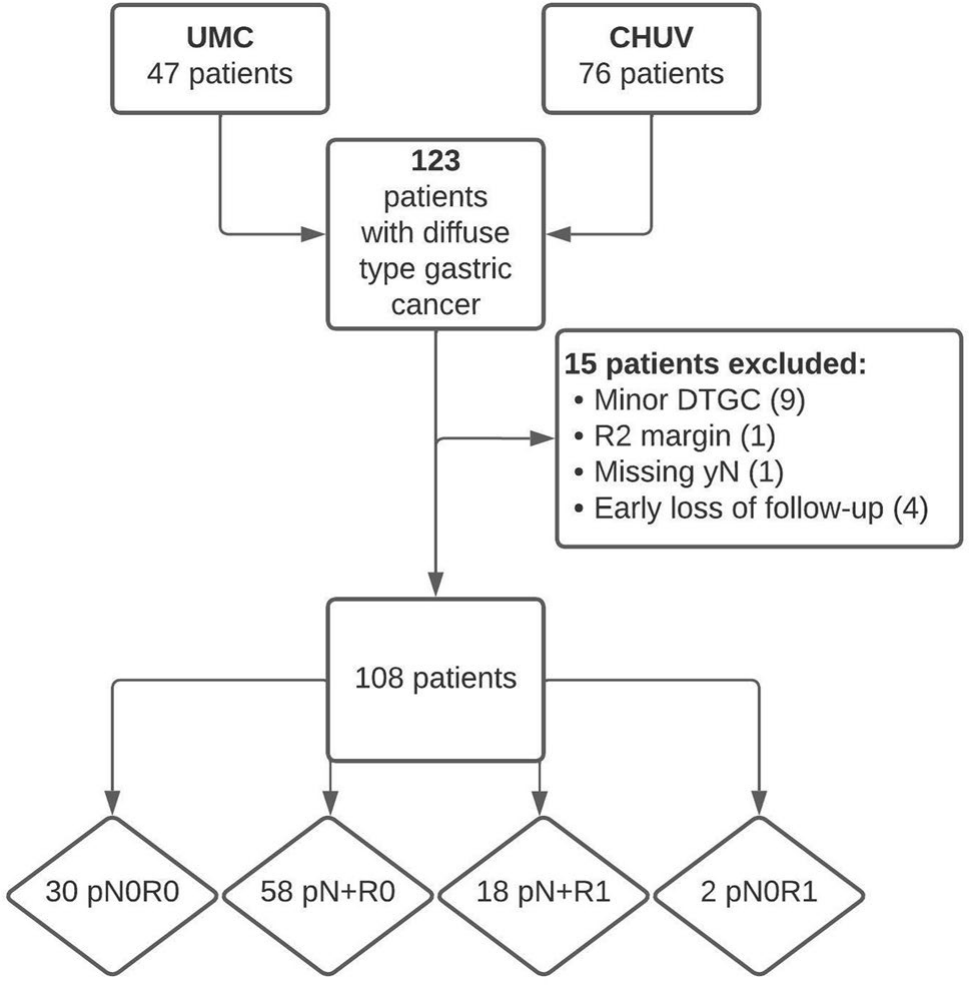
Retrospective cohort selection design and study flow chart. *UMC* Amsterdam UMC (Netherlands), *CHUV* University hospital of Lausanne (Switzerland), *DTGC* diffuse-type gastric cancer, *pN+* > 1 tumour positive in the pathological analysis of lymph nodes, Resection margins (R0, R1): according to the college of American pathologists, *R1* a 0 mm contact between the tumour front and surgical resection margins

Overall, 123 patients with diffuse-type gastric cancer underwent surgery from 2005 to 2018. Of these, 108 patients were finally included in the current analysis, 88 in group R0 and 20 in group R1 respectively (Fig. 1, Flowchart). Baseline patient demographics were similar between both groups (Table 1). Among the 87 patients for whom the cT stage was available, 61% of R0 and 100% of R1 patients had locally advanced (pT3-4) tumours (*p* = 0.002), whereas baseline cN stage was comparable. On the contrary, the pathological T stage was similar in R0 and R1 groups, but R1 patients presented higher rates of positive lymph nodes (N + disease 90% in R1 versus 66% in R0 patients, *p* = 0.046) (Tables 1 and 2). A similar proportion of R0 and R1 patients received preoperative chemotherapy (*p* = 0.447). The extent of gastrectomy (*p* = 0.195) as well as postoperative complications were similar between both groups (*p* = 0.452). Of note, three patients with DTGC had oeso-gastric junction invasion and underwent transthoracic resection with gastric tube reconstruction (Table 2).

**Table 1:** Baseline patient characteristics

	Overall (*N* = 108)	R0 group (*N* = 88)	R1 group (*N* = 20)	*p-*value
Male sex (%)	59 (54)	48 (55)	11 (55)	0.971
Median age (years; IQR)	62 [49–72]	60,5 [48–71]	67 [53–76]	0.139
ASA				
1–2	81 (75)	68 (77)	68 (77)	0.253
3–4	27 (25)	20 (23)	20 (23)	
Baseline cT stage				
1–2	27 (25)	27 (30)	0	**0.002**
3–4	60 (56)	43 (49)	17 (85)	
Missing data	21 (19)	18 (20)	3 (15)	
Baseline cN stage (%)				
0	48 (44)	40 (45)	8 (40)	0.847
1	46 (43)	38 (43)	8 (40)	
2	8 (7)	6 (7)	2 (10)	
Missing data	6 (6)	4 (5)	2 (10)	

Bold values = *p*-value < 0.05

Continuous variables are shown as median [Interquartile range]

Categorical variables are shown as *n* (%)

**Table 2:** Surgical details and postoperative outcomes

	Overall (*N* = 108)	R0 group (*N* = 88)	R1 group (*N* = 20)	*p*-value
Type of gastrectomy (%)				
Ivor-Lewis resection	3 (3)	2 (2)	1 (5)	0.195
Partial gastrectomy	35 (32)	32 (36)	3 (15)	
Total gastrectomy	69 (64)	54 (61)	15 (80)	
Missing data	1 (1)	0	1 (5)	
Minimally invasive surgery	45 (42)	42 (48)	3 (15)	**0.007**
Pre-operative chemotherapy	62 (57)	49 (56)	13 (65)	0.447
Peri-Operative chemotherapy	77 (71)	63 (72)	14 (70)	0.708
Postoperative complications				0.452
Minor I–II	54 (50)	43 (49)	11 (58)	
Major III–IV	44 (41)	37 (42)	7 (37)	
Mortality (V)	2 (2)	1 (1)	1 (5)	
Missing data	8 (7)	7 (8)	1 (5)	
Pathological (y)pT stage				
0	2 (2)	2 (2)	0 (0)	0.268
1–2	36 (34)	32 (36)	4 (20)	
3–4	70 (65)	54 (61)	16 (80)	
Pathological (y)pN stage				
N0	33 (31)	31 (35)	2 (10)	**0.046**
N1	24 (22)	20 (23)	4 (20)	
N2–N3	51(47)	37 (42)	14 (70)	

Bold values = *p*-value < 0.05

Tumor stage was defined by the 8th TNM/UICC staging system

Categorical variables are shown as number (%)

### Survival and recurrence in R1 versus R0 patients

**Fig. 2:**
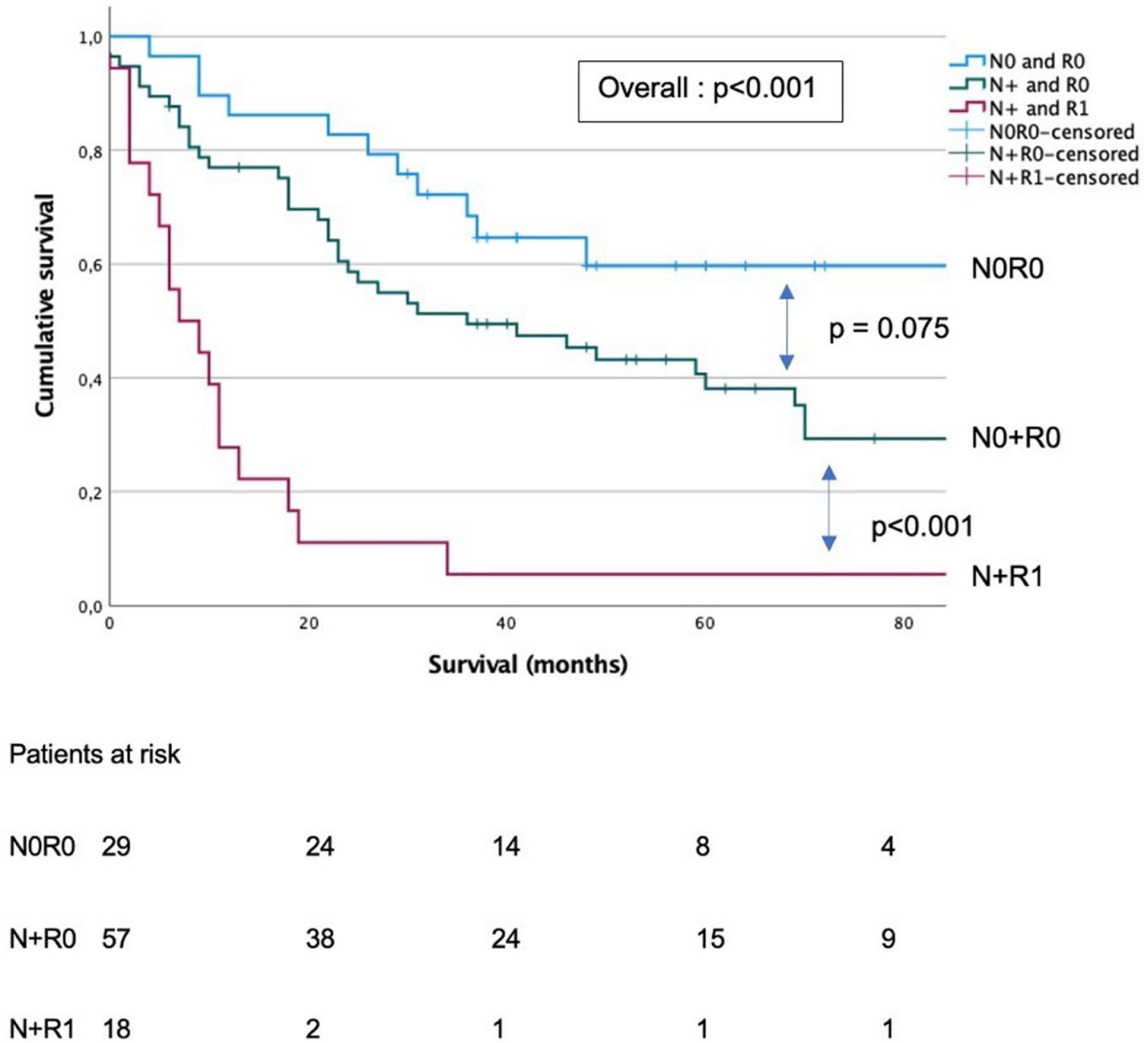
Overall survival for patients with diffuse-type gastric cancer according to their resection margins (R) and lymph node invasion (pN) status. *N0* No tumoral invasion in lymph nodes, *N+*  tumoral invasion in locoregional lymph nodes, *R0* margins resections microscopically free of tumor, *R1* microscopic tumoral involvement of resection margins

Among all included patients, the N0R0 group had the best OS (median 102 months, 95% CI 1–207 months). Patients with lymph node invasion but negative resection margins (pN + R0) had a median OS of 36 months (95% CI 13–59 months), whereas N + R1 patients had a median OS of only 7 months (95% CI 1–13 months, *p* < 0.001). N0R1 group was too small for analysis. Pairwise comparisons were also performed, demonstrating significantly worse survival in R1 patients, even within the pN + group (N0R0 vs N + R0, *p* = 0.075; N0R0 vs N + R1, *p* < 0.001; N + R0 vs N + R1, *p *< 0.001). The pN0R1 subgroup contained only two patients; thus, specific survival analysis was not possible (Fig. 2**)**. On multivariable analysis, (y)pT stage (HR 4.1, 95% CI 2.1–8.0), R status (HR 6.4, 95% CI 3.4–12.3) and perioperative chemotherapy administration (HR 0.4, 95% CI 0.2–0.7) were independently associated with long-term OS (Table 3).

**Table 3:** Cox regression analysis for overall survival and disease-free-survival

	Overall survival (OS)				Disease-free survival (DFS)			
	Unadjusted HR (95% CI)	*p*-value	Adjusted HR (95% CI)	*p*-value	Unadjusted HR (95% CI)	*p*-value	Adjusted HR (95% CI)	*p*-value
ASA class		**0.021**		0.433		**0.046**		0.475
1–2	1 (ref)		1 (ref)		1 (ref)		1 (ref)	
3–4	1.9 (1.1–3.2)		1.2 (0.7–2.2)		1.6 (1–2.6)		1.2 (0.7–1.9)	
(y)pT stage		** < 0.001**		** < 0.001**		** < 0.001**		** < 0.001**
1–2	1 (ref)		1 (ref)		1 (ref)		1 (ref)	
3–4	4.0 (2.1–7.3)		4.1(2.1–8.0)		3.0(1.9–4.7)		3.1 (1.9–4.9)	
(y)pN status		**0.020**		0.843		0.124		
N0	1 (ref)		1 (ref)		1 (ref)			
N +	2.0 (1.1–3.5)		1.1 (0.6–2.0)		1.4 (0.9–2.1)			
R status		** < 0.001**		** < 0.001**		** < 0.001**		** < 0.001**
R0	1 (ref)		1 (ref)		1 (ref)		1 (ref)	
R1	4.7 (2.7–8.3)		6.4 (3.4–12.3)		4.1 (2.4–6.9)		4.7 (2.7–8.2)	
Perioperative CT	0.5 (0.3–0.9)	**0.018**	0.4 (0.2–0.7)	**0.001**	0.8 (0.5–1.2)	0.270		

Bold values = *p*-value < 0.05

*ASA* American Society of Anesthesiologists, *HR* hazard ratio, *CI* confidence interval, *CT* chemotherapy, Tumor stage was defined by the 8th TNM/UICC staging system

**Fig. 3:**
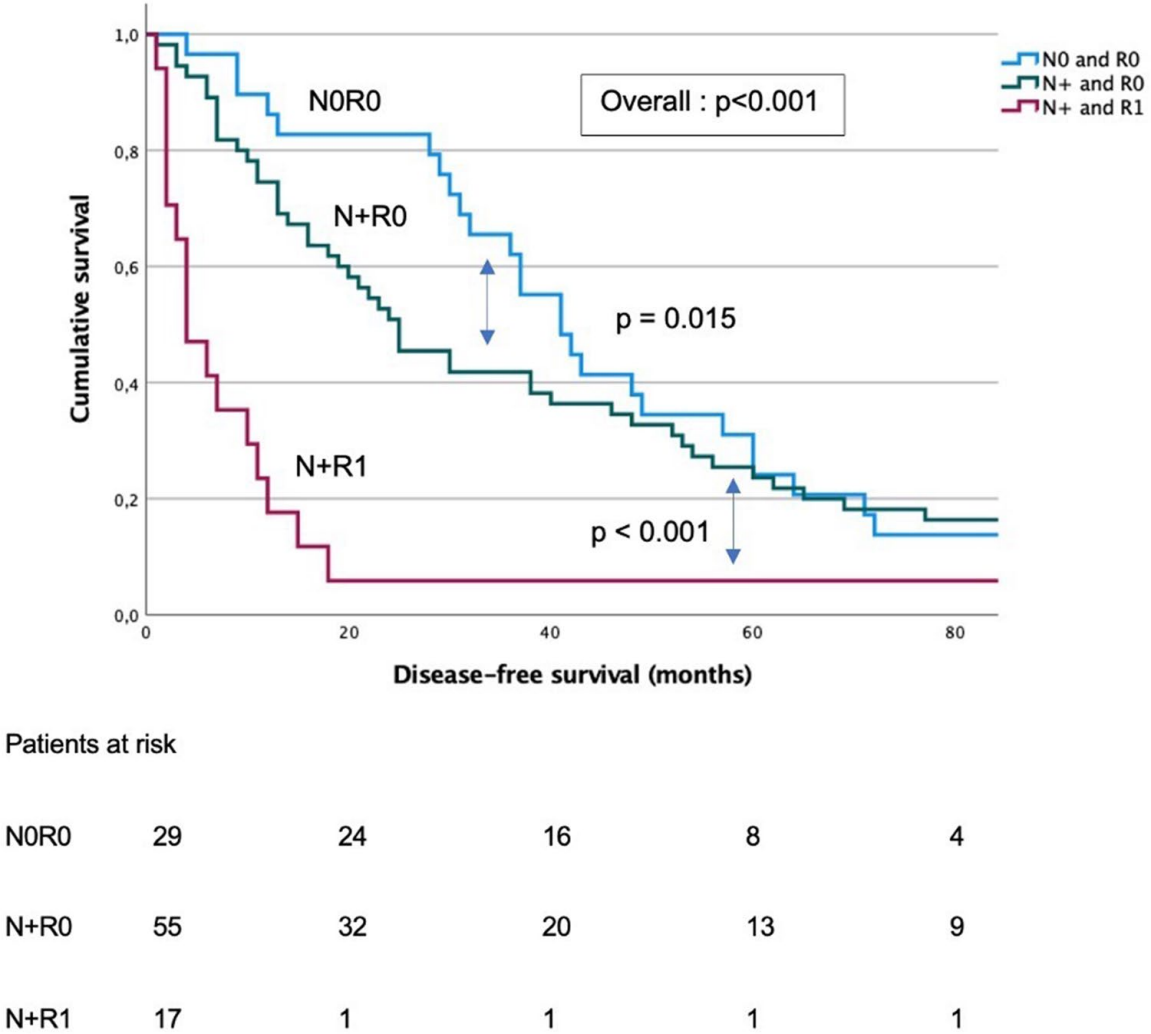
Disease-free survival in patients with diffuse-type gastric cancer according to their resection margins and lymph node invasion status. *N0* No tumoral invasion in lymph nodes, *N+* tumoral invasion in lymph node, *R0* margins resections microscopically free of tumor, *R1* microscopic tumoral involvement of resection margins, *p* *p*-value

Similarly, median DFS was 41 months (95% CI 32–50 months) for pN0R0 patients, 25 months (95% CI 17–33 months) or pN + R0 patients, and 4 months in pN + R1 patients (95% CI 1–7 months) (*p* < 0.001) (Fig. 3). Pairwise analysis illustrated significant inter-group differences (N0R0 vs N + R0, *p* = 0.015; N0R0 vs N + R1, *p* < 0.001; N + R0 vs N + R1, *p* < 0.001). On multivariate analysis only the pT stage (HR 3.1, 95% CI 1.9–4.9) and R status (HR 4.7, 95% CI 2.7–8.2) were independent predictors of DFS (Table 3).

### Recurrence pattern

Among the included 108 patients, 45 (42%) tumour recurrences were recorded during follow-up. Patients with R1 status had more often early recurrence within < 12 months (30% vs 8%, *p* = 0.002) compared to R0 patients. However, no difference was observed in terms of recurrence pattern, and locoregional recurrence rates (22 patients in R0 versus 9 in the R1 group, *p* = 0.14). Logistic regression analysis identified none of the assessed parameters to be independent predictor of locoregional recurrence (Table 4).

**Table 4:** Logistic regression analysis for factors predictive of loco-regional recurrence

	Unadjusted OR (95% CI)	*p*-value	Adjusted OR (95% CI)	*p*-value
ASA		0.414		
1–2	1 (ref)			
3–4	0.7 (0.2–1.8)			
pT stage		**0.036**		0.179
1–2	1 (ref)		1 (ref)	
3–4	2.9 (1.1–7.8)		2.1 (0.7–5.8)	
pN status		**0.026**		0.081
N0	1 (ref)		1 (ref)	
N +	3.7 (1.2–11.6)		2.9 (0.9–9.5)	
R status		0.113		
R0	1 (ref)			
R1	2.2 (0.8–5.8)			
Periop chemotherapy	1.8 (0.7–4.9)	0.254		

Bold values = *p*-value < 0.05

*ASA* American Society of Anesthesiologists status, Tumor stage was defined by the 8th TNM/UICC staging system, *OR* odds ratio, *CI* confidence interval

### Effect of chemotherapy *on survival of diffuse-type gastric cancer patients*

**Fig. 4:**
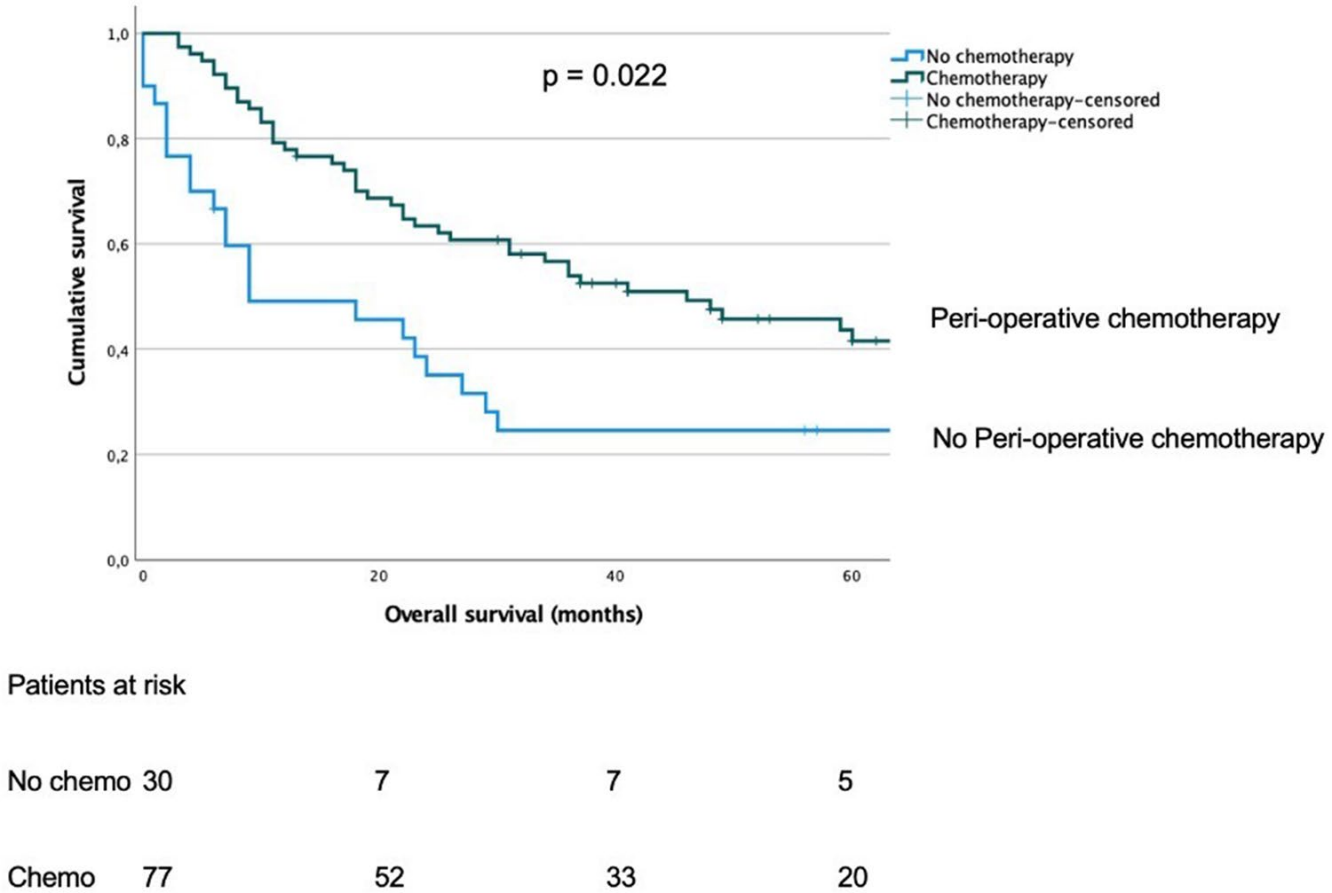
Kaplan–Meier analysis of overall survival for patients with diffuse-type gastric cancer according to per-operative chemotherapy status. *p* p-value

Seventy-seven patients received perioperative chemotherapy. The median OS for patients who received perioperative chemotherapy was 46 months (95% CI 24–68 months) versus 9 months (95% CI 1–25 months) for those who underwent upfront surgery (*p* = 0.022) **(**Fig. 4**)**. Both groups were similar in terms of baseline characteristics (Data not shown). Chemotherapy regimens were 5-FU-platin based, with notable heterogeneity in specific drug combinations even within each centre.

## Discussion

In the present series, assessing 108 patients undergoing oncologic gastrectomy for diffuse-type gastric cancer (DTGC), R1 resection remained significantly associated with poor long-term OS and DFS, even among patients with N + disease. Patients with R1 resection presented higher rates of early systemic recurrence within 12 postoperative months, but not higher rates of locoregional relapse.

### Long-term survival and recurrence depending on resection status

Our study reports that patients with locally advanced (node positive) DTGC had better OS and DFS survival if negative resection margins could be obtained at surgery. The negative impact of R1 resection margins in gastric cancer surgery has been suggested previously by the Dutch Upper gastrointestinal Cancer cohort with a median survival of 23 months for R0 resection and 8 months for R1 resection in 70 patients with DTGC (van der Werf et al. 2019). Adverse prognostic factors were invaded lymph nodes, positive-margin resection and the absence of adjuvant therapy (Stiekema et al. 2013; Pattison et al. 2017). In the present series, on the subgroup of patients with node-positive (pN +) disease, OS was reduced from 17 months in N + R0 patients to 7 months in N + R1 patients. This suggests that even in more advanced disease stage, R1 resection remains a poor prognostic factor for long-term survival. However, apart from surgical margins, other parameters importantly influence long-term survival in these patients, such as overall tumour biology and perioperative systemic treatment.

### Biological behaviour of diffuse-type gastric cancer

Even if DTGC is often considered jointly with the intestinal type in most gastric cancer studies, it is a distinct entity with specific characteristics in epidemiology, molecular pathogenesis, type of dissemination and also response to systemic treatment (Waldum and Fossmark 2018). The initial hypothesis of the present study was that resection margins did not massively influence survival in patients with locally advanced disease, where lymphatic spread was already present. In the same line, Sun et al., in a > 2000 patient series with both diffuse and intestinal types of gastric cancer, reported a loss of prognostic value of R0 resection for stages III–IV (Sun et al. 2009). Several other studies, with positive resection margins ranging from 0.8 to 20%, identify several factors related to R + resection, such as DTGC histology, deep mucosal invasion, tumour size and lymph node invasion (Kim et al. 1999; Cascinu et al. 1999). Bickenbach et al. reported 54% of DTGC vs 29% intestinal type among patients with positive resection margins, all of whom had early-stage disease (Bickenbach et al. 2013). The extensive submucosal spread in DTGC instead of a well-delimited intra-luminal mass seems to account for the higher rates of microscopic margin invasion in diffuse-type lesions, which is why a macroscopic resection margin larger than 5–8 cm for DTGC is recommended whenever possible (Stiekema et al. 2013; Japanese Gastric Cancer Association 2017; Mönig et al. 2020).

### Systemic chemotherapy in diffuse-type gastric cancer patients

During the last two decades, gastric cancer treatment has markedly evolved. The MAGIC trial proved the superiority of chemotherapy and surgery versus surgery alone, offering a 5-year survival benefit of 13.3% (36.3% vs 23%) (Cunningham et al. 2006). More recently, the FLOT regimen was proven to be superior to the ECF regimen for both gastric cancer subtypes, with encouraging results, even for the DTGC subtype (Al-Batran et al. 2019). Few studies tried to compare survival between diffuse and intestinal-type patients after peri-operative chemotherapy. Schirren et al. reported better OS for patients with intestinal or mixed subtypes than those with DTGC treated with perioperative 5-FU/platin-based treatment (Schirren et al. 2021). The biologic aggressiveness of DTGC and its relative chemo-resistance has led to the development of several new chemotherapy options, mostly studied among Asian populations, that seem to offer a OS benefit for DTGC patients (e.g. S-1 fluoropyrimidine derivate and capecitabine) (Takahari et al. 2014; Ma et al. 2016; Pattison et al. 2017; Al-Batran et al. 2019).

In the present series, patients with DTGC demonstrated a significant survival benefit after peri-operative chemotherapy, even though chemotherapy regimens were heterogenous in both participating centres and most of the patients were operated before the introduction of the FLOT regimen. These results suggest that patients with DTGC, should receive systemic treatment to improve long-term outcomes, whenever their clinical and general health status allows it (Smyth et al. 2016).

### Recurrence patterns in diffuse-type gastric cancer

In the present series, 45 patients (42%) presented tumour relapse during follow-up. Interestingly, this recurrence was loco-regional in only a minority of cases (28.7% of all). These results are somewhat contradictory with previously published studies, suggesting a predilection for loco-regional recurrence in DTGC (Lee et al. 2018). R1 resection is often perceived as a risk factor for local ‘failure, and the question of re-operation or postoperative radiotherapy rises for these patients. Cho et al. reported loco-regional recurrence in 40% of patients but only suggested re-operation in fit patients with the early-stage disease with negative lymph nodes (Cho et al. 2007). In addition, the latest treatment guidelines suggest a clear survival benefit for postoperative chemotherapy, but not an established role for radiotherapy in this context (Lordick et al. 2022). Stessin et al. suggested an OS advantage for patients who undergo postoperative radiotherapy, but the correlation with positive margins was not specifically assessed (Stessin et al. 2014). In contrast to previous reports, the present study suggests an increased risk for early (< 12 months after surgery) metastatic recurrence in R1 DTGC patients, but no correlation between R1 margins and an increased risk of local recurrence. These results support the need for a close postoperative follow-up in this high-risk patient group, but no expected benefit from an adjuvant local treatment due to R1 margins. Although a clear physiopathological explanation cannot be provided for the decreased risk of local relapse in our series compared to previous literature, meticulous surgical technique, and the high rates of preoperative chemotherapy use (57% in the whole series) might potentially contribute to the low rates of local failure, by limiting the risk of intra-operative tumour spillage. This important point needs further validation in future studies, as nowadays local intraperitoneal chemotherapy (HIPEC, PIPAC) could be offered in high-risk patient groups (Yonemura et al. 2006; Alyami et al. 2019; Brenkman et al. 2019). Ongoing trials like the PREVENT study are currently assessing the impact of HIPEC in DTGC (Götze et al. 2021).

The present study has some limitations that need to be discussed. First, gastric cancer and especially DTGC histology remains infrequent in European populations, thus the sample size remains small, even with the participation of two tertiary referral centres. In addition, the long period of patient inclusion introduces some heterogeneity in perioperative management and chemotherapy protocols, especially with the recent introduction of the FLOT regimen. Despite this, the here presented real-life data suggests may be regarded as further insight into this rare disease and its prognostic factors, especially in Western population where large homogenous series are difficult to obtain. Lastly, the retrospective character of the study is closely related to the problem of missing data. As provided data were anonymized by both centres according to the Ethics Committee decisions, returning to patients’ files to complete the missing data was impossible.

## Conclusion

In the present series of patients with diffuse-type gastric cancer, positive gastric margins (R1) after gastrectomy were an independent negative prognostic factor of overall survival and disease-free survival, in both node-negative and node-positive disease. R1 resection was associated with an increased risk of early recurrence but not with and increased risk of local recurrence compared to R0 patients. Perioperative chemotherapy offered survival benefits.

## Data Availability

The datasets generated during and/or analysed during the current study are available from the corresponding author on reasonable request.
